# Universal Stochastic Multiscale Image Fusion: An Example Application for Shale Rock

**DOI:** 10.1038/srep15880

**Published:** 2015-11-02

**Authors:** Kirill M. Gerke, Marina V. Karsanina, Dirk Mallants

**Affiliations:** 1 CSIRO Land and Water, Glen Osmond, PB2, SA 5064, Australia; 2 The University of Melbourne, Department of Infrastructure Engineering, Parkville, VIC, 3010, Australia; 3 Institute of Geosphere Dynamics of the Russian Academy of Sciences, Leninsky prosp. 38/1, Moscow, 119334, Russia; 4 Institute of Physics of the Earth of Russian Academy of Sciences, Bolshaya Gruzinskaya 10, Moscow, 107031, Russia

## Abstract

Spatial data captured with sensors of different resolution would provide a maximum degree of information if the data were to be merged into a single image representing all scales. We develop a general solution for merging multiscale categorical spatial data into a single dataset using stochastic reconstructions with rescaled correlation functions. The versatility of the method is demonstrated by merging three images of shale rock representing macro, micro and nanoscale spatial information on mineral, organic matter and porosity distribution. Merging multiscale images of shale rock is pivotal to quantify more reliably petrophysical properties needed for production optimization and environmental impacts minimization. Images obtained by X-ray microtomography and scanning electron microscopy were fused into a single image with predefined resolution. The methodology is sufficiently generic for implementation of other stochastic reconstruction techniques, any number of scales, any number of material phases, and any number of images for a given scale. The methodology can be further used to assess effective properties of fused porous media images or to compress voluminous spatial datasets for efficient data storage. Practical applications are not limited to petroleum engineering or more broadly geosciences, but will also find their way in material sciences, climatology, and remote sensing.

Assembling spatial information across a wide range of scales is a crucial component in almost any type of industrial or scientific activity^1^. Spatial information – discrete property values with known coordinates – is usually reported by means of digital images where values are represented by intensities and coordinates are marked within a grid with known spatial sizes or image resolution (pixels for 2D images and voxels for 3D). Image resolution - a key attribute of all spatial data - has been increasing considerably in all scientific fields. For instance, performance of computed tomography has doubled approximately every two years since the mid 1980s^2^. Likewise, electron microscopes (SEM) typically have a 250–300 times higher resolution than optical microscopes, while the highest spatial resolution of aerial imagery is up to 2.5 cm compared to 50 cm for satellite images. These differences in resolution by no means suggest that lower-resolution methods need to be abandoned; one of their advantages is their much wider field of view or measurement support and, thus, their ability to resolve larger objects than the more recent high-resolution devices. This becomes especially relevant for 3D imaging methods such as X-ray micro-tomography (XMT), focused ion beam–scanning electron microscopy (FIB-SEM) or neutron tomography, where the very small measurement support of the device is prohibitive of determining relevant properties across many locations of the study object. The duality between a sufficiently large, in terms of a representative elementary volume (REV), sample size and a sufficient spatial resolution can be resolved by studying the same object at different spatial scales, followed by upscaling and/or downscaling information into a single image capturing data from all scales.

Merging information across multiple scales is of interest to many scientific disciplines; some meaningful applications relate to (non-exhaustive list): 1) images of outcrops displaying large-scale features of sedimentary units, medium-scale sedimentological facies heterogeneity and fault zones, and small-scale fractures^3^,^4^ to assemble multiscale information on fracture size and connectivity distribution for simulations of flow and transport; 2) large-scale 2/3D seismic images and 1D high-resolution well-logging supported by small-scale core measurements of fluid properties to create realistic oil/gas reservoir models^5^ for improving resolution of reservoir characteristics and properties upscaling schemes; 3) medical imaging^6^ for better diagnostics; 4) merging hydrogeological parameters from aquifer pumping tests, tracer tests and undisturbed core measurements;^7^,^8^ 5) fusing micrometer scale XMT and nanometer scale FIB-SEM images to derive effective properties such as apparent permeability or gas desorption rates of low-permeable carbonates, shales and coal seams^9^ using pore-scale modelling approaches;^10^ 6) combining satellite images with plot-scale and point-scale on-ground measurements of plant species distributions, CO_2_ or CH_4_ emissions, or soil moisture conditions^11^,^12^.

Unlike time-domain and multi-sensor imaging (i.e., merging of images of the same object obtained with different imaging techniques, for example, in different wavelengths) where substantial progress was made by means of Fourier transform and wavelets^1^, the spatial domain yet lacks any comprehensive methodology to unify information across scales. Previous work regarding merging spatial information usually considered a single type of data, often measured by a single image, with very few material phases. Radlinski *et al.*^13^. combined SEM images and small-angle neutron scattering (SANS) data within a statistical framework to quantify the structure of porous solid in terms of continuous pore-size distribution spanning five orders of magnitude. A similar approach was used to characterize pore structure and permeability of sandstone samples using combined information from thin-sections and mercury intrusion porosimetry^14^. Bimodal porous structures were previously created by superimposing two truncated Gaussian random fields to simulate vuggy carbonates^15^. A similar methodology was also recently utilized to synthesize layered structures by connecting two separate images^16^. These bi-gaussian approaches can be considered a special case of the more general pluri-gaussian method popular in geostatistics. Previous works further include multiscale structures using truncated pluri-gaussian simulations^17^,^18^, however mathematically elegant, this approach utilizes only a limited number of statistical descriptors and requires tuning. Okabe and Blunt^19^,^20^ developed a general approach to a 2/3D reconstruction of the structure of porous solids via multiple-point statistics in a multi-grid framework using 2D cuts of sandstones. A slightly different Bayesian based approach was reported by Mohebi *et al.*^21^. to reconstruct double porosity features of different artificial and natural porous media samples using coarse and high resolution 2D magnetic resonance images. Continuum reconstruction of Fontainebleau sandstone was performed using pre-defined grain shapes based on multiscale digitization of sphere pack^22^. Other groups of researchers^23^,^24^ modelled carbonate rock structures with pore-sizes varying in size up to four orders of magnitude using SEM/thin-section and XMT data; their approach considered a primordial filter (static data from coarse scale image) decorated with crystal-filled molds of different size typical of dolomite structure. Merging two different scales of pore sizes in rocks was recently demonstrated using pore-network generation^25^,^26^,^27^.

The aim of this study is to develop a general framework for merging information from any number of spatial scales of any resolution into a single image, which would be simple, robust, and efficient. The methodology will be tested using structural images from shale rocks, for which data fusion will be performed using images at three spatial scales representing different spatial information on the distribution of mineral, organic matter and pore phases. Our results are expected to have practical implications in numerous disciplines, e.g., petroleum engineering, geosciences, material sciences, hydrology, soil science, biology, climatology/ecology, and remote sensing.

## Multiscale Fusion of Shale Images

### Defining the test problems

The multiscale data fusion method will be demonstrated on the basis of two synthetic test problems. However synthetic, these tests are aiming to solve one of the long standing challenges in petroleum geosciences, i.e. to fuse or merge structure data from macro, micro and nanoscale into a single, multiscale structure of shale rock. Shales are the source rock of shale gas and shale oil, and are known to have multiscale pore systems, composed of macroscale fractures, micropores, and nanoscale pores within gas or oil-producing organic material, referred to as kerogen. The former two scales are visible on XMT images, but are usually not connected into a percolation network^28^. The nanoscale pore network is organized in different interconnected clusters and extends through mudrocks where desorption and diffusion occurs within the kerogen with subsequent transport into larger-scale micropores and fractures produced by hydraulic fracturing^29^,^30^,^31^. Fractures may be naturally present or produced during hydraulic fracturing. XMT can efficiently resolve the 3D geometries of fractures and microscale pores within shale rocks, but cannot capture nanoporosity^28^. Higher-resolution two-dimensional imaging techniques such as SEM or FIB-SEM are effective to discern the pore network down to scales of several nm^32^. Such pore-networks find their application in the simulation of oil and gas flow during design and operation of hydrocarbon reservoirs, using so-called pore-scale models that produce effective physical properties for use in larger scale cellular Darcy-scale models^28^,^29^. To this end, the macro, micro and nanoscale pore-system have to be merged into a single network continuum with proper connections between scales.

**Figure 1:**
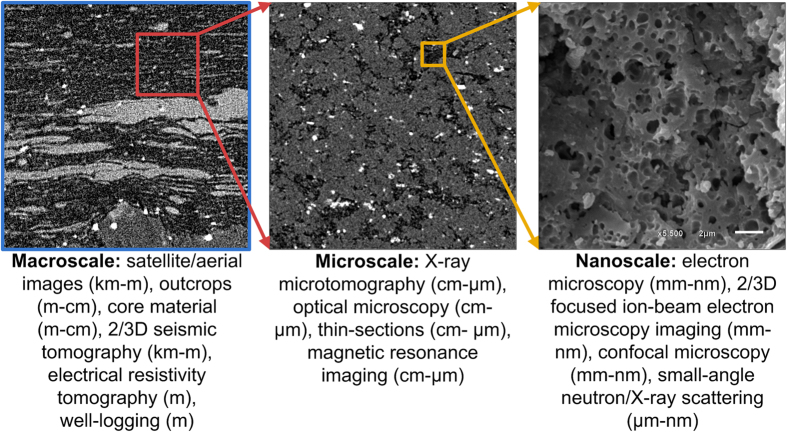
Three images of shale structure representing macro, micro and nanoscale (from left to right). Below each image we report some common methods to obtain spatial information for widely differing scales. Typical values for field of view and resolution are given in parentheses.

Three different images of shale structure are shown on Fig. 1 comprising macroscale, microscale and nanoscale. Images represent shale samples from Domanic (macroscale) and Upper Jurasic Bazhenov (micro and nanoscale) formations, respectively from the Tatar Basin and West Siberian Basin, Russia (see brief description in SI). The Bazhenov formation generated about 90 percent of oil reserves of the West Siberian basin^33^. The microscale image displays basically the same solids and kerogen phase as the macroscale, whereas the porosity now excludes the fractures (Fig. 1). Finally, the nanoscale image provides the kerogen nanoporosity.

Both examples consider merging three multiscale images with scale ratios of 16:4:1. In other words, the macroscale is four times coarser than the microscale, which in turn is four times lower in resolution than the nanoscale image. For reasons of visibility, a relatively small scale magnification factor of four was used. Although the real images used in Fig. 1 have a different scale ratio than 16:4:1, this does not preclude their use in our synthetic examples. The original image sizes were 1024^2^ pixels for both macro and microscale and 400^2^ for nanoscale. Each image is first segmented into a binary image of white and black phases representing different rock phases (SI Fig. 1). We consider two cases in which unique binary phases (i.e. materials) exist at each spatial resolution. In the first case, the macroscale image represents spatial information on solids or minerals (white phase) and a mixture of microscale solids not identifiable on the macroscale and kerogen (black phase). The latter phase is resolved on the microscale image in terms of minerals (white) and poorly defined kerogen (black) phases. Finally, the nanoscale image has a sufficient resolution to discriminate the kerogen into kerogen solids (white) and kerogen porosity (black). This is a common situation where each spatial scale represents unique information on one of the material phases due to a large contrast in resolution or size of structural features. For example, kerogen nanoporosity can be resolved only on FIB-SEM/SEM images, while on XMT images kerogen is visible only as a single phase representing a mixture with other materials^28^.

The second case is about merging multiscale pore networks while using exactly the same input images. The pore network information includes 1) large cracks (white) and a mixture of solids and kerogen (black) on the microscale image, 2) microscale porosity (black) and kerogen phase (white) on the microscale image, and 3) similar to first case, kerogen solids (white) and kerogen porosity (black) on the nanoscale image.

Both cases represent a simplification of the real shale structure, mainly because the number of relevant phases is reduced in our study^28^. However, it provides us with an easily comprehensible illustration utilizing only binary information for each scale. By considering the resolution of XMT to be 1 μm and representative for the microscale, the image resolutions are fixed as follows: 4 μm for macroscale, 1 μm for microscale and 250 nm for the nanoscale image. Note that such resolutions are quite typical for routine rock imaging, as resolutions of 4–15 μm are usually obtained for standardized petrophysical cylinders used for laboratory measurements of permeability and other filtration properties^10^. Resolutions of 0.7–1 μm are the usual resolution for desktop scanners with cylindrical subdrilled sample with diameter of 4–10 mm^28^. The highest resolution is obtained with SEM images, which typically is in the range of several tens of nm^32^.

### Re-scaling correlation functions and stochastic reconstructions

The backbone of the data fusion approach lies in the use of stochastic reconstruction^34^,^35^, a mathematical technique that produces a statistically similar representation of the real image and possess similar statistical properties. Previous examples have usually used the same image from one or all scales^1^,^21^,^23^,^24^, an approach that limits the size of reconstructed images or requires to use tiling (replicating similar image by connecting its copies of itself). Tiling is not useful for natural porous media as natural properties are not periodic and, thus, phases would not meet each other at the edges of tiles. Stochastic reconstructions using correlation functions is a versatile technique^36^ capable of dealing with any image size and with a wide range of complex structural descriptors. In addition to geometrical complexity, the image size is another important factor as it determines whether or not the image is large enough to capture all spatial features accurately.

**Figure 2:**
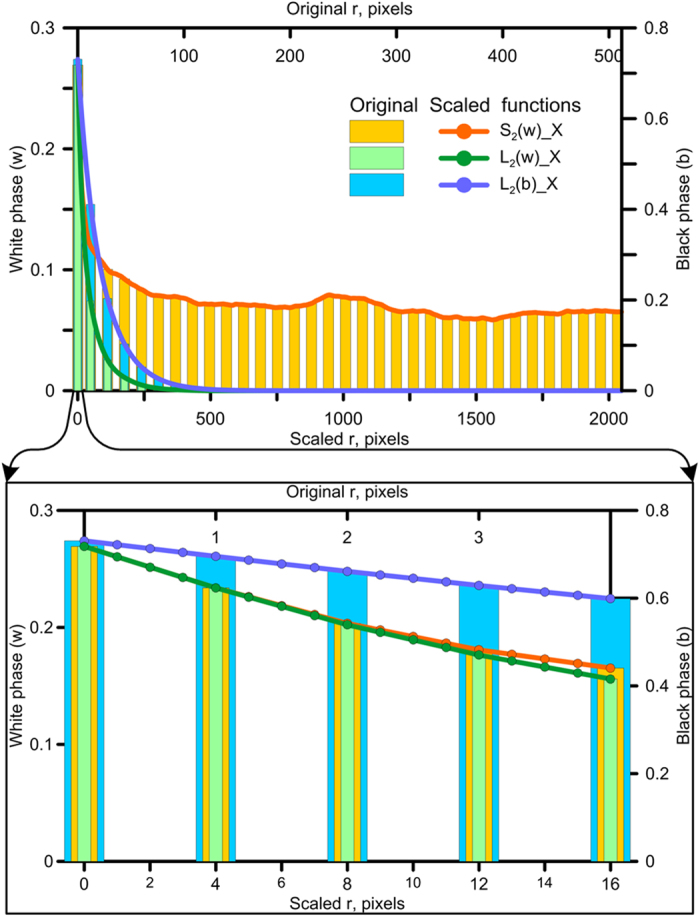
Example of four times downscaled correlation functions for macroscale image. Inset shows a magnification of the beginning part of the functions.

Among many stochastic reconstruction techniques applicable to heterogeneous porous media, the more popular ones are: (i) the original method of Yeong-Torquato, combining correlation functions with simulated annealing^35^, (ii) the multiple-point statistics method^19^,^20^,^37^,^38^ and (iii) Gaussian random fields^39^. The modified Yeong-Torquato method was chosen here for reconstruction of all three scales because (i) of its ability to reproduce both isotropic and anisotropic porous media^40^ and (ii) the correlation functions can be rescaled to accommodate either smaller or larger scales. Spatial two-point correlation functions can take different forms (e.g. linear, cluster) and are calculated as probabilities of different image events or patterns related to a line segment of given length ***r***^41^. An important feature of the correlation functions is the ability to be scaled, by which the resolution of the original correlation function can be upscaled or downscaled (Fig. 2a). Upscaling of two-point probability function dates back to the 90s^34^ and was routinely performed to coarsen the Guassian random field reconstructions to minimize scarce computational and memory resources of that time. Later on, both upscaling and downscaling of hypothetical two-point probability functions were used to create synthetic bimodal structures^15^,^16^. Rescaling of different types of directional correlation functions calculated from multiscale images of real porous media as part of the Yeong-Torquato stochastic reconstructions has, to the best of our knowledge, not been previously reported. Linear ***r*** interpolation is used for downscaling the macroscale image producing an increased resolution (a factor of four in Fig. 2b); a similar approach was used for upscaling the correlation function resulting in a coarsening of the resolution (SI Fig. 6). One of the main advantages of rescaling correlation functions is the ability to stochastically reconstruct images originally different in resolution into images with identical resolution, i.e., representing a super-resolution process for binary image^42^. This approach does not require any assumptions about unresolved spatial information (e.g., about fractal characteristics^43^). The latter means that smooth interpolation does not necessarily produce similar results compared to correlation functions calculated directly on the magnified image. Nevertheless, this is exactly what we want at this stage, as higher resolution details are implemented later. Downscaling and upscaling by interpolation provides reasonable accuracy for our images, as was demonstrated in a comparison between reconstruction tests on some simple images involving both rescaled correlation functions and direct image scaling (see SI for details).

**Figure 3:**
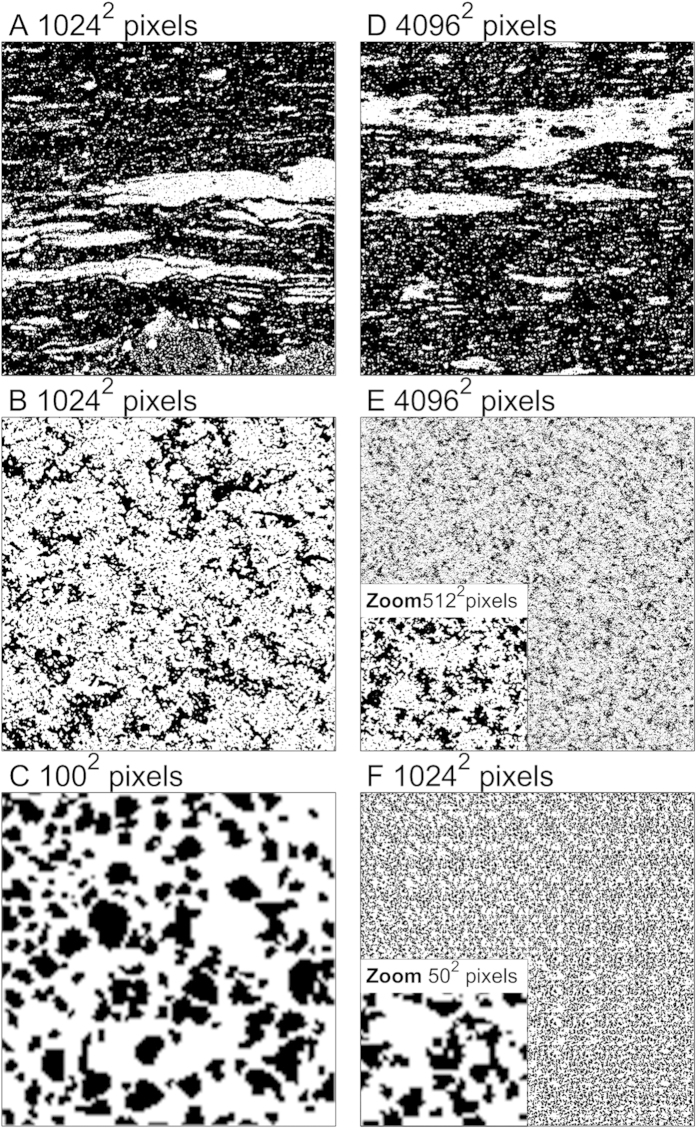
Original segmented images (a–c) and corresponding stochastic reconstructions (d–f) used for image fusion. Original macroscale (**a**), microscale (**b**), and nanoscale (**c**) image. Reconstructed macroscale (**d**), microscale (**e**), and nanoscale (**f**) image. Insets show magnified area to compare with original image.

The reconstruction procedure involves developing all images (three in the current example) with the same resolution: our example considers 1 μm resolution, the intermediate of three scales, to demonstrate both up and downscaling procedures. In theory, images of identical resolution can be based on any of the original scales, although the smallest-scale resolution would be preferred. Downscaling the correlation functions for the 4 μm resolution macroscale image four times produces a reconstructed image of 1 μm resolution at the microscale. To illustrate the applicability of the method to other reconstruction techniques such as multiple-point statistics, simple coarsening of the images is applied for nanoscale image before reconstruction rather than rescaling the correlation functions (the original nanoscale image resolution was coarsened four times by image rescaling, i.e., from 400^2^ to 100^2^ pixels, which produced a relatively similar reconstruction as based on a set of rescaled correlation functions with some loss of porosity, see examples and discussion in SI Fig. 7–9). Three images of equal size (4096^2^ pixels) are generated with a resolution of 1 μm based on correlation functions for macro, micro and nanoscale images. For reasons discussed in SI (SI. 2 and SI Fig. 5), nanoscale image tiling was used, which involved a reconstructed 1024^2^ pixel image that was multiplied four times to obtain a 4096^2^ pixel image. By applying periodic boundary conditions during stochastic reconstruction, tiling produces smooth images without edge connection problems. Input images and resulting stochastic reconstructions are shown in Fig. 3 (separate images are also available in SI). Methodological details about stochastic reconstructions are covered in the Materials and Methods section.

### Merging multiscale images

**Figure 4:**
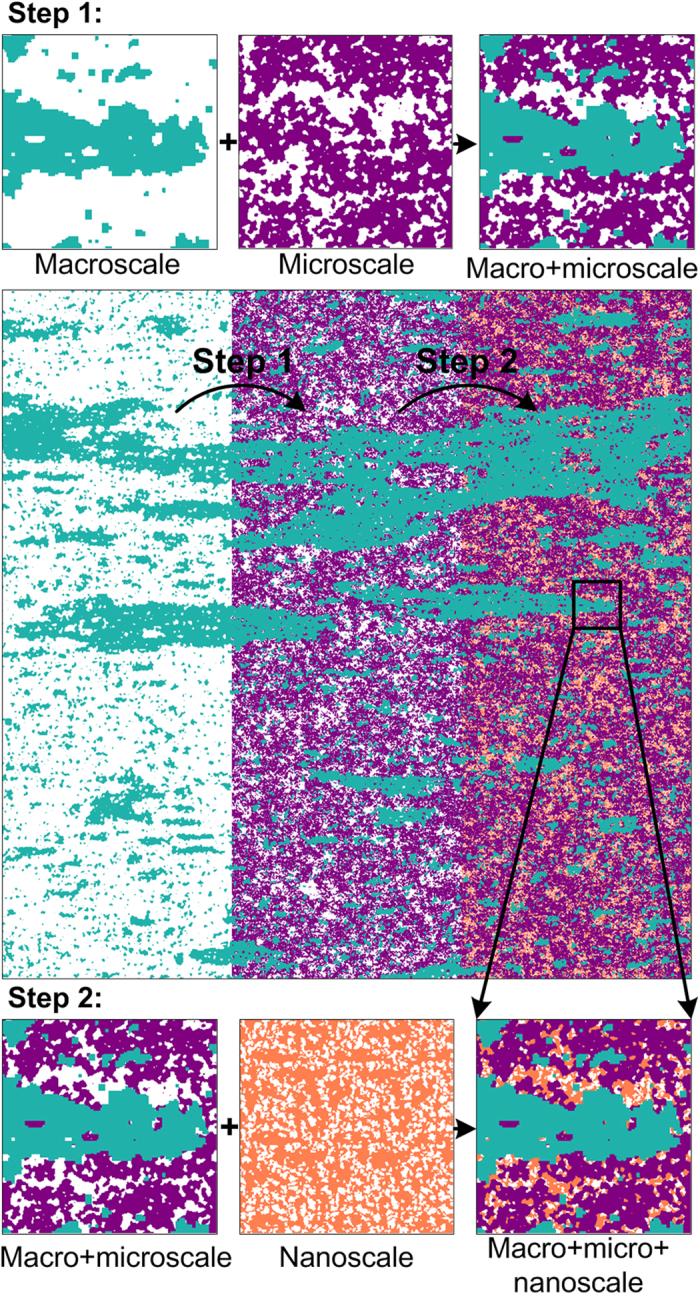
A step by step embedding of three phases (minerals, kerogen, and pores) by fusion of three stochastic reconstructions. Phases are combined as follows: step 1 combines (**i**) resolved mineral (blue) and unresolved non-mineral phase (white) on macroscale image plus (**ii**) mixture of resolved minerals and kerogen phases (purple) and unresolved kerogen with nano-pores (white) on microscale image; step 2 combines the output of step 1 plus (**iii**) resolved kerogen (organic matter) phase (orange) and resolved kerogen nano-porosity (white) on nanoscale image.

**Figure 5:**
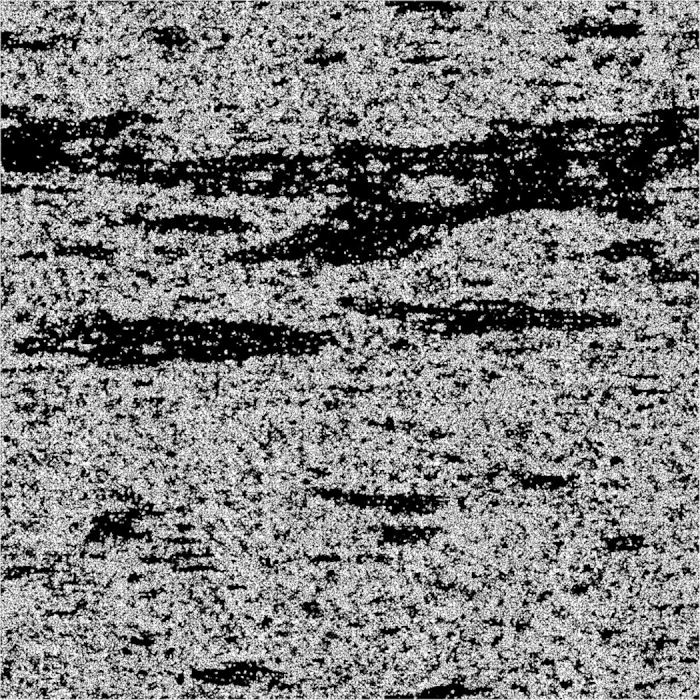
Multiscale pore network obtained from fusing three different scales representing macrocracks, microscale and nanoscale porosity. Merging is performed in similar manner as on Fig. 4; porosity is shown in black, while white represents solids.

The overall workflow schematic for merging spatial information from macro, micro and nanoscale images is shown in SI Fig. 10. Once images of similar size and resolution have been created from rescaled correlation functions, the workflow proceeds with the actual merging step. This involves a step by step embedding of all phases according to the following sequence (based on the shale rock data for the first case). The example considers a two-step infilling of the non-mineral phase (white coloured phase in Fig. 4); in theory merging scales could also be applied to the mineral phase if subsequent higher resolution images exist with identification of different minerals. At first, we merge macro and microscale information by substituting mineral and kerogen phases (purple on Fig. 4) into the white phase on the macroscale image to obtain the combined macro-microscale image. Step two involves merging the previously obtained image with nanoscale information: the kerogen structure is substituted (orange on Fig. 4) into the white phase. Note that the white phase before each infilling step represents a phase which is not properly spatially resolved and, thus, requires information substitution from a higher-resolution imaging method. In summary, embedding is performed by overlaying three stochastic reconstruction images with decreasing scale (increasing resolution) whereby the mineral phase (blue on the macroscale and purple on the microscale) is opaque and the non-mineral phase (white) is transparent for the next higher-resolution scale (Fig. 4). For the second case of multiscale porosity, the merging procedure is similar, except that white and black phases are treated differently. The final fused image (Fig. 5) is shown in binary way to clearly highlight the multiscale pore network (shown in black) and solids (white).

While the overall methodology is similar for both examples, there is a difference in the way images are merged and phases combined. The first example is specifically designed for applications where a spatial arrangement of numerous phases, materials or values is important: e.g., for 1) multiphase materials such as composites^41^,^44^, 2) soil moisture, carbon fluxes or vegetation maps^11^,^12^, 3) porosity, permeability and other hydraulic properties distributions^5^,^7^,^8^,^45^. Our second example highlights the problem of merging multiscale pore-networks that can be used for pore-scale modelling of effective properties (such as apparent and relative permeabilities^10^,^25^,^26^,^27^,^28^,^29^) or utilized for flow and transport simulations in highly heterogeneous formations^3^,^4^,^46^,^47^. An example involving both approaches can be important for modelling shale gas production rates, or for multiphase flow in mixed-wet systems where knowledge of not only the pore-network itself, but also of pore forming material is crucial to properly quantify desorption rates or menisci configurations^10^,^30^. All full size fused images from these examples are available as 4096^2^ pixels tiff files and are included into SI.

## Discussion and Outlook

The methodological framework for merging spatial information from different scales into a single dataset has many potential applications. The relevance of this method to earth sciences was illustrated by solving the long standing challenge of combining multiscale structural information for shale rock. The backbone of our method is based on stochastic reconstructions using rescaled correlation functions. This stochastic approach is especially useful in case high-resolution data cannot be obtained for the entire object under study, a case that exists for shales as FIB-SEM/SEM imaging is too laborious to be performed within the whole volume of the rock sample. However, for reasons of display clarity, the test problem was solved in 2D only, although the extension to 3D is straightforward^48^. Note that 3D problems can be solved using only a set of 2D images (cross-perpendicular 2D cuts for anisotropic 3D structures) as input data, thus highlighting another very important advantage of the stochastic approach. It is important to mention that for porous media applications such as the shale example considered here, 3D realization of our technique with real scale ratios will result in large 3D images with over a billion of voxels. With recent advancements in CFD/pore-scale modelling on the core-scale volumes^49^,^50^, our methodology is now also very appealing for upscaling of porous media properties, or for verifying other approaches such as averaging/homogenization^51^,^52^, pore-network generation^25^,^26^,^27^, or continuum Darcy-scale simulations^28^,^46^,^47^.

Other advantages of the data fusing method include: (i) provided that input data is representative for all given scales and phases, merging can be performed using limited data (i.e. a single REV size image for each embedded phase suffices), (ii) a combination of any practical number of scales and/or images can be performed, and (iii) for each of the final phases, the resulting image is statistically similar to the original images. Rescaling of correlation functions for obtaining a multiscale image has several additional benefits (based on the shale rock example): (i) it keeps phase ratios constant for any rescaling, and (ii) it does not produce “pixelized” cubical objects during downscaling (SI Fig. 7). The rescaling reconstruction can be further refined by incorporating information from higher-resolution images for cross-correlation with lower-resolution images or by performing rescaling using so-called basis functions^44^,^53^.

At this stage the abilities of the proposed method are limited mainly by 1) computational resources (as merging scales of higher resolution contrasts requires stochastic reconstructions of larger images), and 2) accuracy of the stochastic reconstructions. The first limitation can be effectively resolved by incorporating hierarchical stochastic reconstruction methods^54^,^55^, implementing some means of parallelization^56^ and more sophisticated pixel/voxel interchange strategies^55^,^57^, or by using tiling. Details on computing resource specifications and the computation times for the current example are provided in SI (section SI. 6). Reconstruction accuracy can be further improved by using a more comprehensive set of correlation functions to better capture image information content, as at present no universal method exists^58^,^59^. A detailed discussion on current issues related to reconstruction accuracy was recently published^60^ and is not reported here. It was proven^15^ that an intact autocorrelation (two-point probability) function for a superimposed bimodal structure is the same as for a fully resolved image. This leads us to conclude that the accuracy of our merging methodology should depend only on the accuracy of the stochastic reconstruction. The main drawback of the current realization is that cross-correlations between different phases during merging are not taken into consideration. This can be an issue if non-stationarities or transition zones are present at border regions between different phases (i.e., correlation functions for a material close to an interface is different from the bulk phase). Such information is not available in our synthetic dataset, as interfaces between embedded phases are not resolved on images (e.g., the kerogen-minerals interface is not captured on an SEM image) and requires more research. If better input data can be obtained, cross-correlations can be effectively implemented during merging procedures as a separate simulated annealing cycle. The same approach can be used for “stitching” together tiles obtained from rescaling and reconstruction of multiple input images derived from the same spatial scale.

Having numerous advantages, correlation functions are not the only stochastic reconstruction methods. As mentioned earlier, other techniques include 1) Gaussian random fields^39^, 2) multiple-point statistics^19^,^20^,^37^,^38^, 3) entropic descriptors^61^, 4) morphological algorithms^23^,^24^, and 5) process-based or grain algorithms^62^,^63^. Multiple-point methods, for instance, can be easily implemented here by scaling all input images to a fixed resolution, as was done with the nanoscale image in our test problem. Many possibilities exist to hybridize our approach. For example, for sedimentary rocks, multiple-point methods can be used for the macroscale, process-based methods can be used for the microscale and correlation functions for the nanoscale. Irrespective of the reconstruction method implemented, any number of phases can be implemented by multiphase segmentation^64^. For example, in our shale rock test case, microscale pores and chalcedony porosity in the mineral phase could be incorporated^28^. The basic idea of embedding information into different phases is shown in SI Fig. 11. This shows that however the realization of merging (or superimposing) in current examples is similar to that in the pluri-gaussian methodology, it is actually more flexible and allows for more diverse superpositioning. Tiling (or other modifications) can be also implemented at this stage to incorporate information from different images of the same phase, e.g. numerous SEM images of kerogen with different pore structure and porosity values^28^. Different degrees of determinism can be employed during fusing as, for example, if the coarsest information available covers the whole object under study a direct structure can be used during merging instead of stochastic reconstruction (SI Fig. 12). The number of phases and reconstruction accuracy can also be significantly increased by multiphase reconstruction methods^45^,^55^,^65^.

In addition to applications in the aforementioned scientific fields, the multiscale data fusion methodology can help resolve a number of other fundamental challenges, such as: 1) to study and determine REVs^66^ of physical parameters used (coupled with pore-scale modelling) in large-scale simulation models of flow, mass transfer and electromagnetic or seismic wave propagation, 2) to reconstruct statistically inhomogeneous random media^67^ by sequential reconstruction and subsequent merging of homogeneous parts, and 3) to compress voluminous images by representing them as numerous sets of correlation functions allowing reconstruction on a short notice as an efficient way of data storage and retrieval.

## Materials and Methods

To stochastically reconstruct each single-scale image for a given size we utilize different sets of (rescaled) directional correlation functions^40^,^68^ computed from original binary images of real rocks. This involves the Yeong-Torquato technique^35^ based on simulated annealing optimization^69^. In this study three types of correlations functions are employed: (i) the two-point probability function S_2_ describing the probability that two points separated by a vector displacement 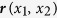 between *x*_*1*_ and *x*_*2*_ lie in the same phase, (ii) the linear function L_2_ describing the probability that the whole segment ***r*** lies within the given phase, and (iii) the two-point cluster function C_2_ describing the probability that two point separated by ***r*** lie in the same cluster. Reconstructions using S_2_ alone are known to be inaccurate due to the numerous degeneracy states and insufficient information content^59^. Simultaneous use of S_2_ and L_2_ was shown to improve reconstruction quality significantly^70^ and addition of C_2_ was effective in solving connectivity issues^36^,^44^. It is well known that two-point probability functions do not discriminate between binary phases, this means that one cannot improve statistical information for a given structure by computing both 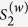 and 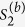 (superscript refers to the binary phase, which is either white or black). The L_2_ and C_2_ functions, unlike two-point probability functions, do discriminate between phases and can be utilized simultaneously to increase information content of the correlation function sets used for the reconstruction procedure. We calculate S_2_, L_2_ and C_2_ functions in two orthogonal and two diagonal directions, which were then used separately during reconstruction^40^.

Provided with correlation functions, we reconstruct spatial structure using the Yeong-Torquato technique, which tries to match correlation functions of a given realization with an original reference structure by pixel permutations. If a set of two-point correlation functions used in reconstruction is provided in the form of 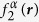, where *α* is a type of correlation function and ***r*** is a segment of varying length, the difference between two realizations of the structure can be expressed as the sum of squared differences between sets of correlation functions:^35^,^40^,^68^


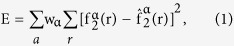


where 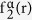 and 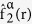 are the values of the correlation function sets for two realizations (where the former represents a reference structure while the latter represents the structure under reconstruction), w_*α*_ is a weighting factor used to improve convergence. In Eq. 1 E represents the “energy” of the system, which is minimized by the simulated annealing algorithm. Initially, we create a random structure and start to change pixel positions, while checking the system’s energy according to Eq. 1. Because at the beginning of this process the characteristic sizes of phase aggregates are typically smaller than for the original image, it is reasonable to accept more permutations even if they do not reduce E. For the purpose of allowing greater flexibility at the initial stage of the inverse modelling scheme, a cooling schedule is invoked for the simulated annealing algorithm which describes the probability of accepting any permutation *p* in the following way:


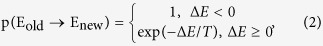


where T is the so-called “temperature” of the system, as interpreted from the Boltzmann distribution used in the lower part of Eq. 2, and


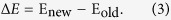


The initial temperature T is chosen so that the probability p for 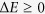 equals 0.5^48^. The idea behind the cooling schedule is that simulated annealing will result in a global minimum of energy E, and would not get stuck in some local minima. From numerous trials and experience in reconstructing different test cases, we chose a slightly slow cooling schedule based on geometrical progression of the form:


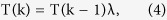


where *k* is time step and λ is a parameter smaller but close to unity.

To accelerate simulations, we optimize recalculation of correlation functions to avoid recalculating the whole set after each pixel permutation. For this purpose we used a very efficient purpose-built optimization algorithm developed for S_2_ and L_2_ functions; a similar approach has not yet been developed for the C_2_ function. Although some speed-up improvements for cluster functions have been reported^44^, reconstructions based on small domain sizes suggests that they are still less efficient compared to S_2_ and L_2_ functions. Also, instead of random permutations, we adopted a relatively simple permutation approach following ref. 70. This involved (i) choosing a random location within a phase of interest, and (ii) choosing two random directions in which two pairs of pixels with a minimum distance in-between are selected such that they satisfy the conditions of lying in opposite phases and at the interface. Finally, further reduction in computational effort can be based on limiting the length of segments used for calculating correlation functions for the reconstruction procedure, i.e. by applying a cut-off to 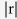.

Periodic boundary conditions were applied for correlation function evaluation during both the reference set evaluation and reconstruction procedure. The reconstruction procedure was terminated after 10^6^ consecutive unsuccessful permutations. We used the annealing schedule parameter λ = 0.999999 for all reconstructions. Weighting factors w_α_ were chosen according to the recently proposed methodology^71^. Rescaling was performed only for the macroscale image as explain in Fig. 2b by taking averages between adjustment points. During the reconstruction procedure each direction for each function is included separately in Eq. 1. Following sets of correlation functions were used: 
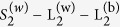
 for macroscale and microscale images (evaluated in four directions, resulting in a total of 12 correlation functions), and 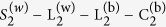 for the nanoscale image (resulting in a total of 16 functions). Details about correlation function sets as well as resulting full images for each scale can be found in SI Fig. 2–5.

Macro and microscale images were binarized by the indicator kriging segmentation method^72^,^73^; the nanoscale SEM image was segmented as described in ref. 28.

## Additional Information

**How to cite this article**: Gerke, K. M. *et al.* Universal Stochastic Multiscale Image Fusion: An Example Application for Shale Rock. *Sci. Rep.* **5**, 15880; doi: 10.1038/srep15880 (2015).

## Supplementary Material

Supplementary Information
