# Supplementary material for: Universal Stochastic Multiscale Image Fusion: An Example Application for Shale Rock
Source: Sci Rep. 2015 Nov 2;5:15880. doi: 10.1038/srep15880 (PMC4629112; doi:10.1038/srep15880)
Supplement: Supplementary Information [file srep15880-s1.pdf]

Supplementary information

"Universal Stochastic Multiscale Image Fusion: An Example Application for Shale Rock"

by Kirill M. Gerke[1,2,3,4*+]**, Marina V. Karsanina**[1,3,4+]**, Dirk Mallants**[1]

[1]CSIRO Land and Water, Glen Osmond, PB2, SA 5064, Australia

[2]The University of Melbourne, Department of Infrastructure Engineering, Parkville, VIC, 3010, Australia

[3]Institute of Geosphere Dynamics of the Russian Academy of Sciences, Leninsky prosp. 38/1, Moscow, 119334, Russia

[4]Institute of Physics of the Earth of Russian Academy of Sciences, Bolshaya Gruzinskaya 10, Moscow, 107031,Russia

corresponding author information: Kirill Gerke, e-mail: kirill.gerke@gmail.com

SI1. Description of macro, micro and nanoscale images. As mentioned in the main text, our images for the test problems originate from different geological formations. They were chosen to represent hypothetical multi-scale images. Macro-scale and micro-scale images were obtained by X-ray microtomography with resolution of 1.2 µm, representing Domanic[1] and Bazhenov[2-3] formation shales. The nanoscale image was obtained for the kerogen phase in Bazhenov shale using SEM with a resolution of 12 nm[3]. Images were subcropped and re-scaled (only nanoscale image), and segmented as described in the Materials and methods section. All original images and subsequent image processing steps are shown in SIFig.1.

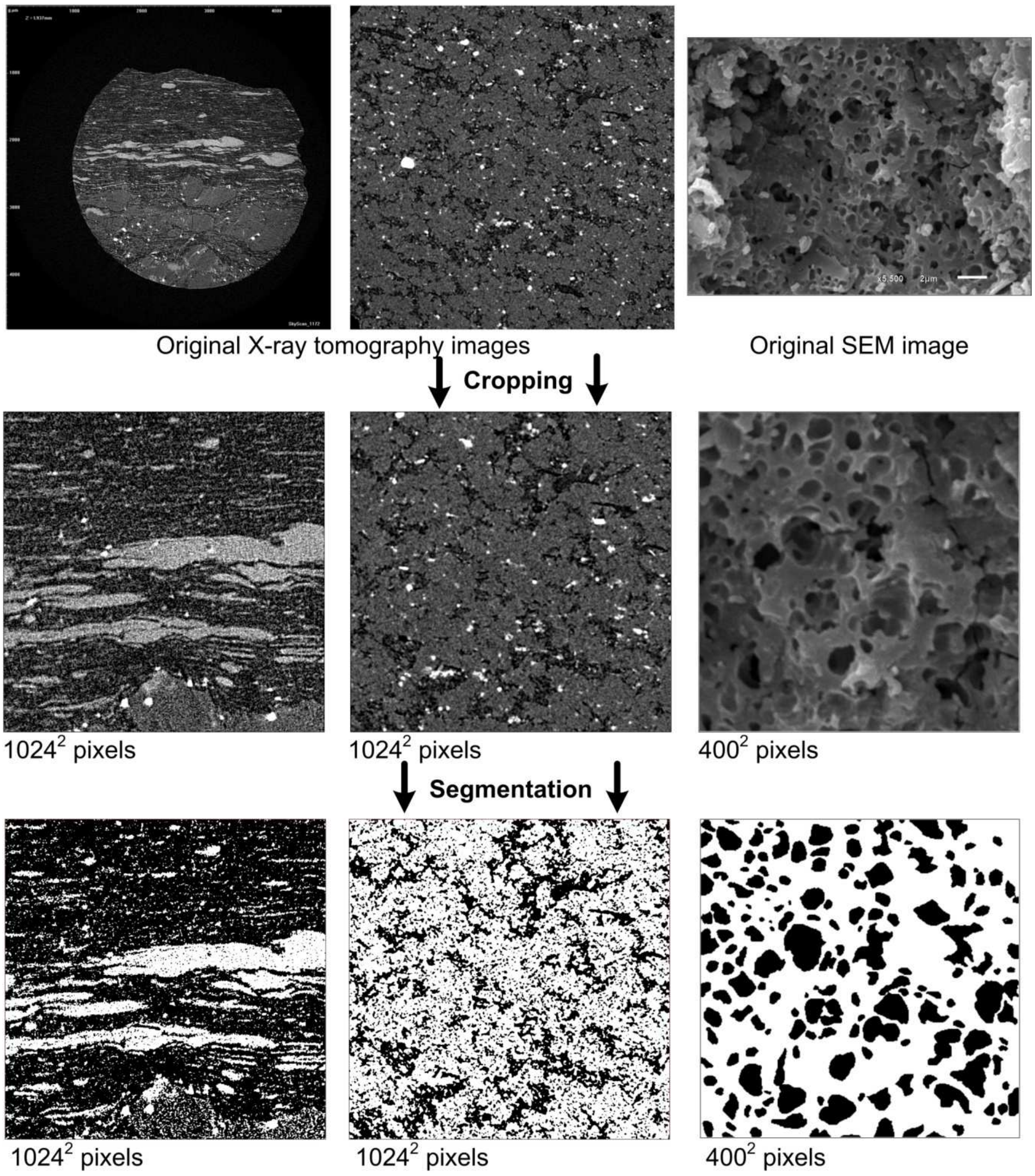

SI Figure 1 Original images of Domanic and Bazhenov formation shales obtained using X-ray microtomography, and kerogen SEM image in Bazhenov shales. Subsequent subcropped and segmented images are shown below each original image.

SI2. Correlation function sets for each binary image. In this subsection we present all correlation functions used for stochastic reconstructions (SIFig.2-5). They were calculated closely following previous work[4] and the Materials and methods section. We implemented cluster functions for the nanoscale image, because the use of only two-point and linear functions for reconstruction resulted in a too high connectivity of the black phase (pores in kerogen); implementation of the C_2 function significantly improved reconstruction quality (SIFig.2D).

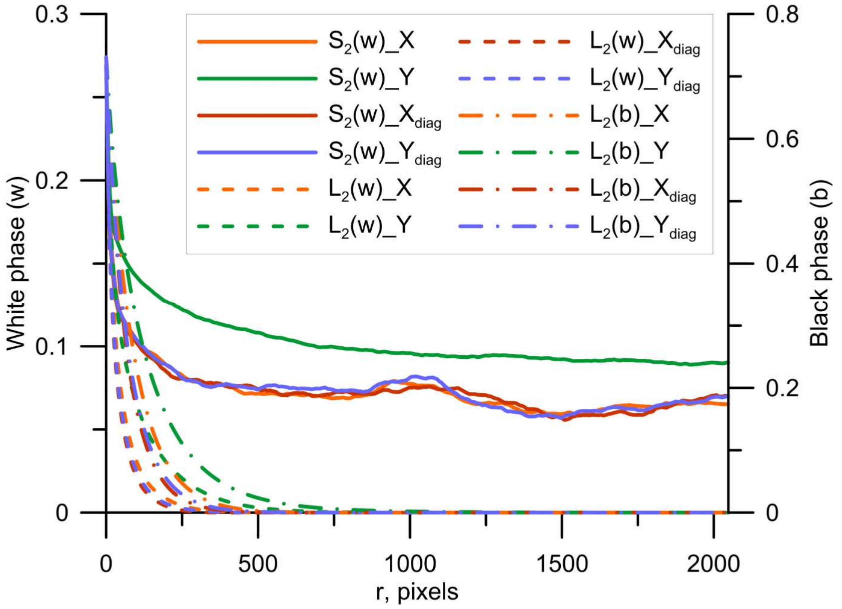

SI Figure 2. Correlation functions used for reconstruction of the macroscale image (after rescaling of correlation functions to improve resolution in four times).

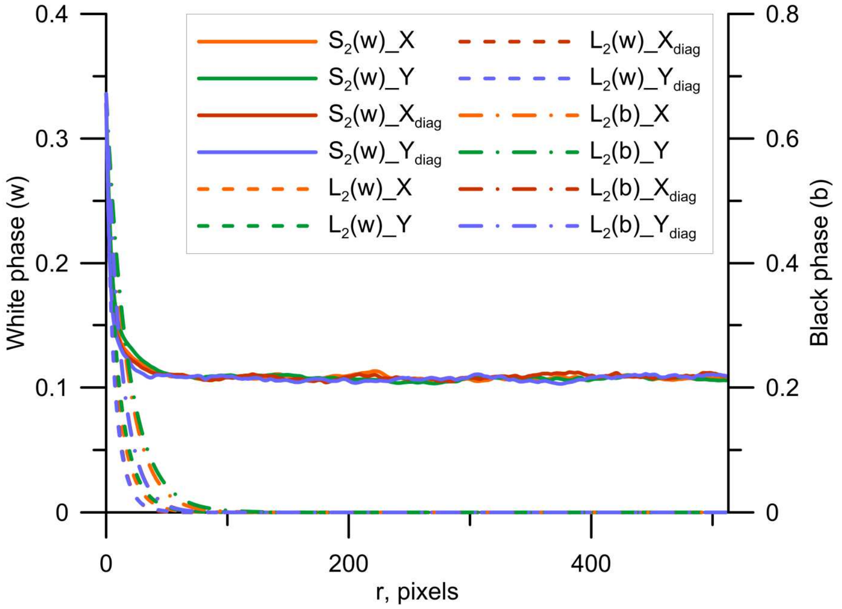

SI Figure 3. Correlation functions used for reconstruction of the microscale image.

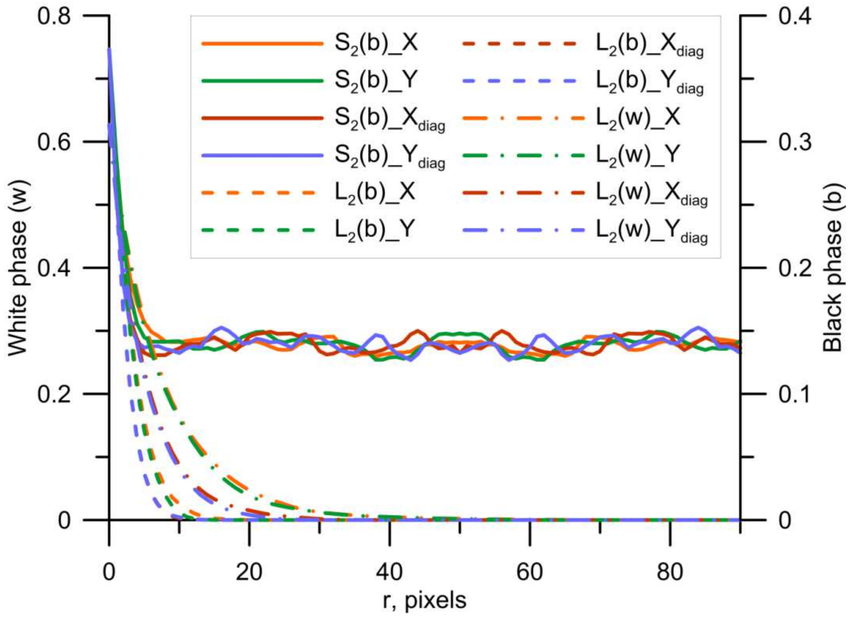

SI Figure 4. S_2 and L_2 correlation functions used for reconstruction of the nanoscale image.

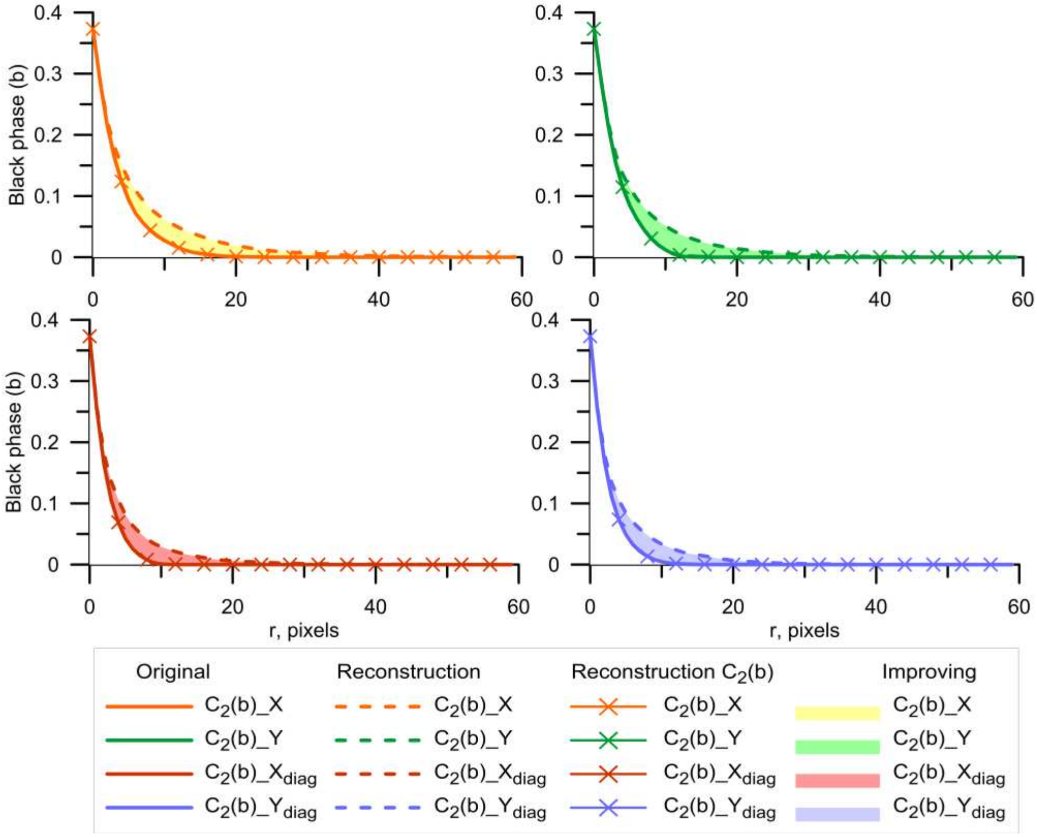

SI Figure 5. Cluster C_2 correlation functions used to improve the reconstruction of the nanoscale image: (left images) reconstruction based on only S_2-L_2, (right images) improved reconstruction using S_2- L_2-C_2. Implementation of the C_2 function resulted in pore connectivity similar to that of the original nanoporosity image. Black phase is kerogen porosity.

SI3. Reconstructions using rescaled correlation functions: examples on simple binary structures. To ensure that rescaling of correlation functions results in proper reconstructions (similar enlarged structure with the same phase fraction's ratio), we conduct tests on two simply binary structures: circles and crosses. We choose these periodic structures as they can be reconstructed exactly[4-5] and thus represent visually efficient test cases. Both structures were properly reconstructed in magnified versions, as demonstrated in SIFig.6.

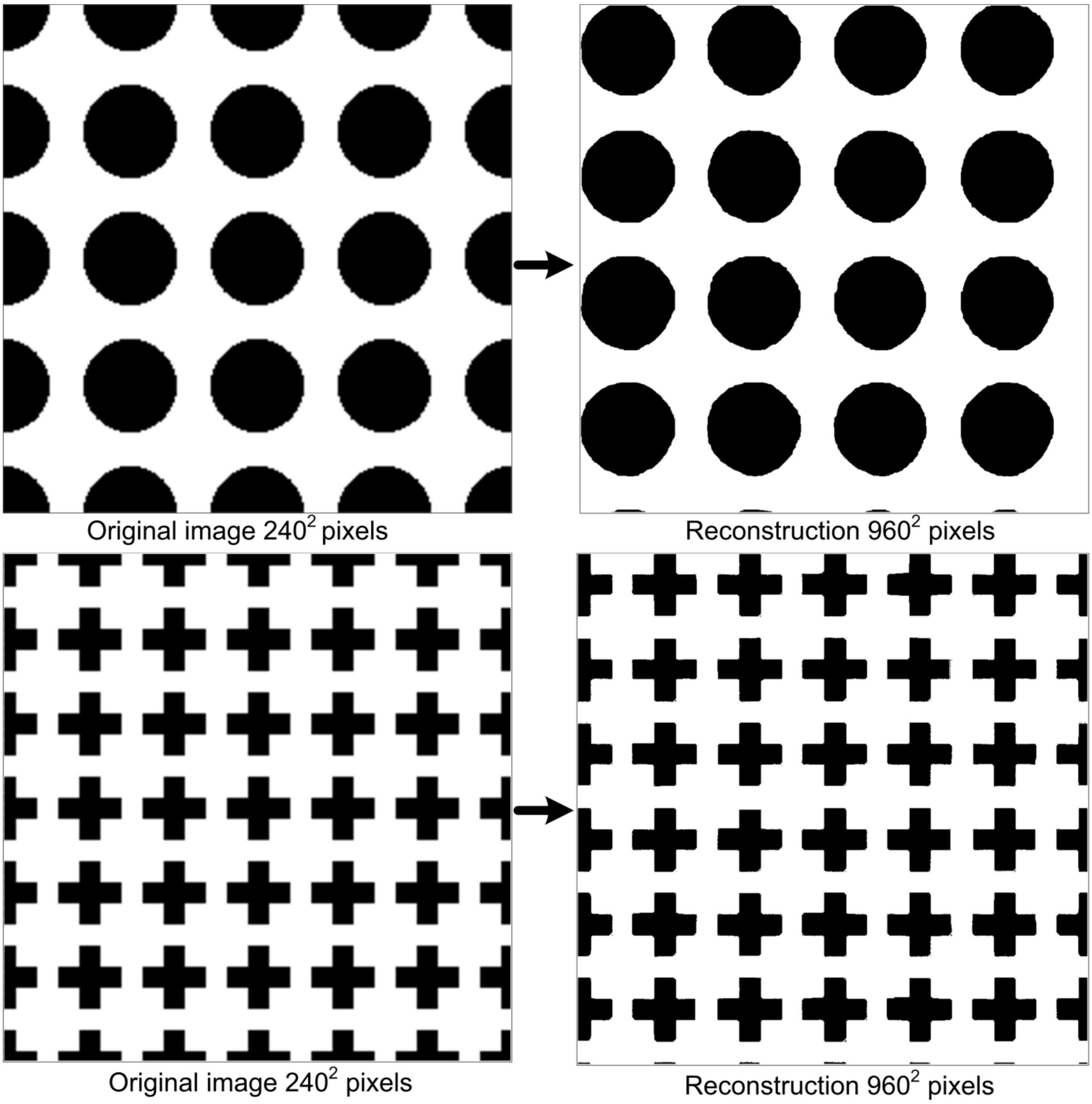

SI Figure 6 Reconstructions of circles and crosses with four times downscaled (with improved resolution) correlation functions.

SI4. Comparison of direct image rescaling and rescaled correlation function reconstruction. To illustrate the difference between direct image scaling and reconstructions using rescaled correlation functions, we compare upscaled and downscaled versions resulting from utilizing these two different approaches (SIFig.7). It is evident that downscaling using correlation functions results in smooth edges, while for image rescaling we observe "pixelized" structure. For upscaling we observe similar patterns, however, both direct up and downscaling of images changes white/black phases ratio, while this is not an issue with correlation functions.

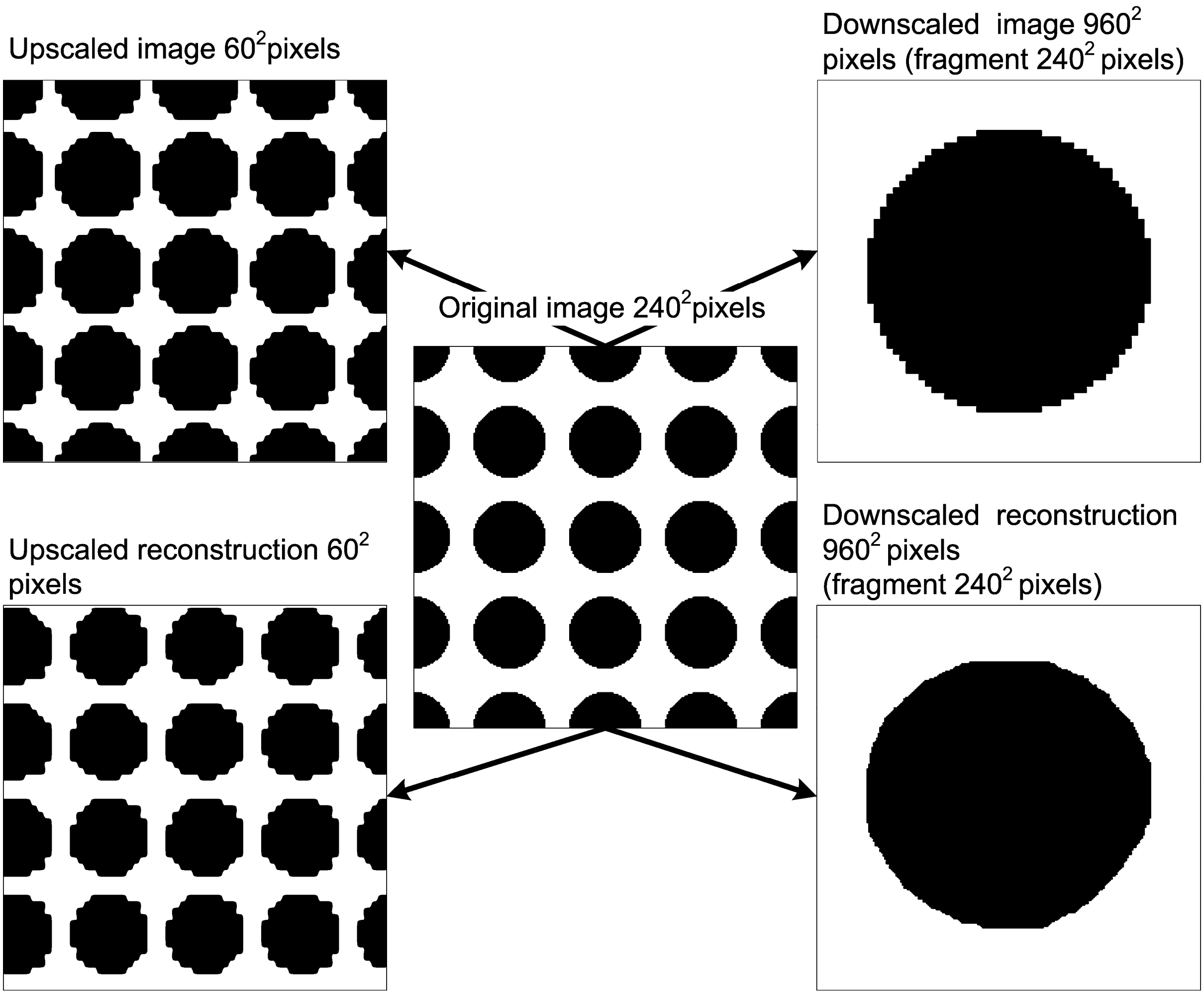

SI Figure 7 Comparison of image qualities obtained for direct scaling of circles and by means of reconstructions using rescaled correlation functions.

To further elucidate the quality of rescaled correlation functions and superiority of this approach to change scales we now compare rescaled correlation functions with correlation functions of rescaled images. Circles from SIFig.7 were chosen for this comparison. Simultaneous visualization of these correlation function sets resulted in almost similar curves, thus preventing any comparison. For this reason, instead of simply putting correlation functions on a single graph, we quantitatively compare 1:4 upscaling and downscaling procedures by diagrams showing the calculated difference between corresponding correlation

functions for rescaled image and rescaled correlation functions for the original image (SIFig.7). Obtained results are presented in SIFig.8 and SIFig.9.

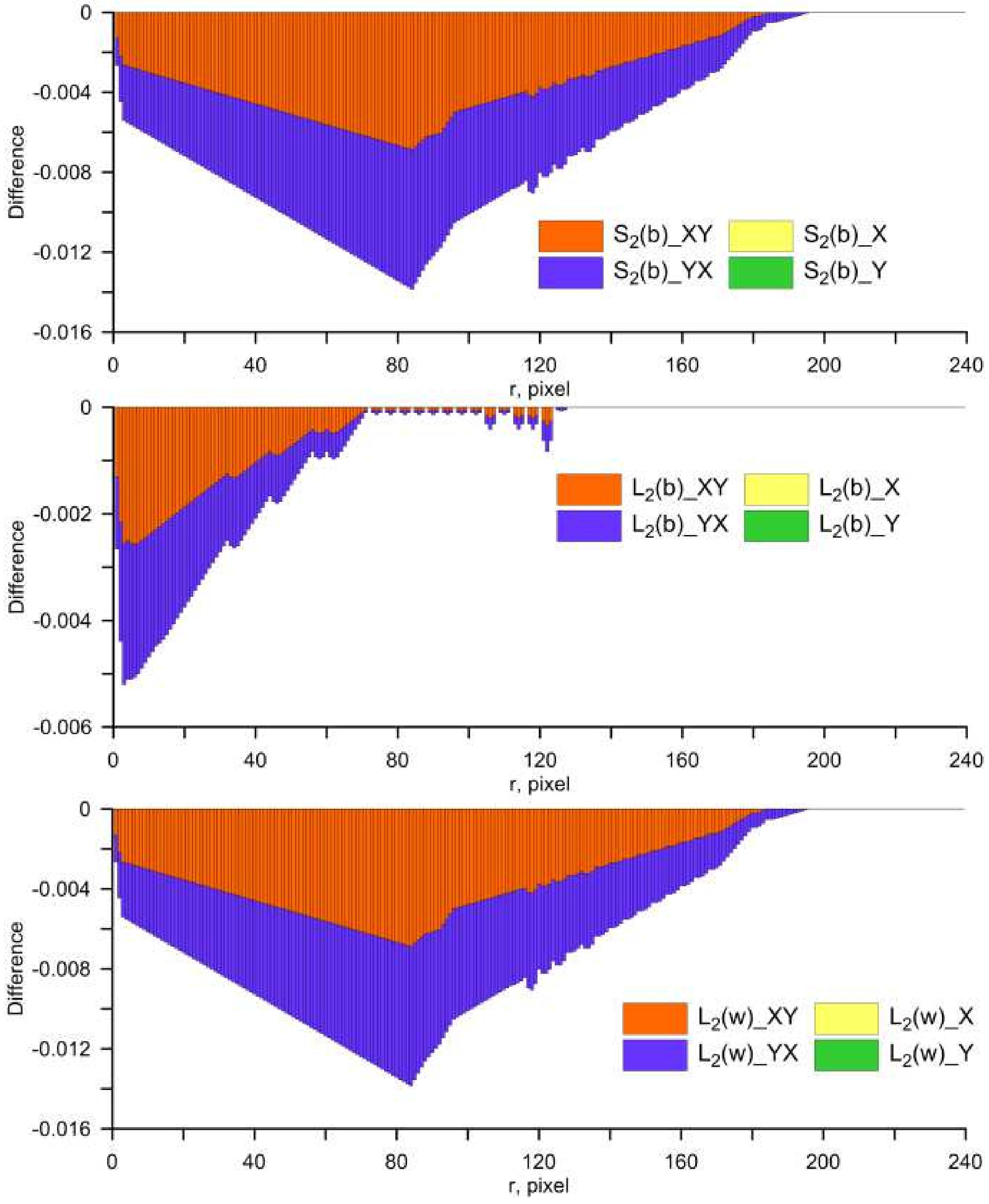

SI Figure 8 Difference between S_2-L_2 correlation functions computed for downscaled (improved resolution) image and downscaled correlation functions for original image (the latter is substituted from the former for each directional segment length ***r***). From top to bottom differences are shown for S_2, L_2 for black and L_2 for white phase. As expected differences exist only in diagonal directions, as direct 1:4 image rescaling simply magnifies each original pixel four times creating discrepancies in diagonal directions ("pixelized" step-like non-smooth edges). Both correlation functions preserve original phase fraction (0th moment of each correlation function).

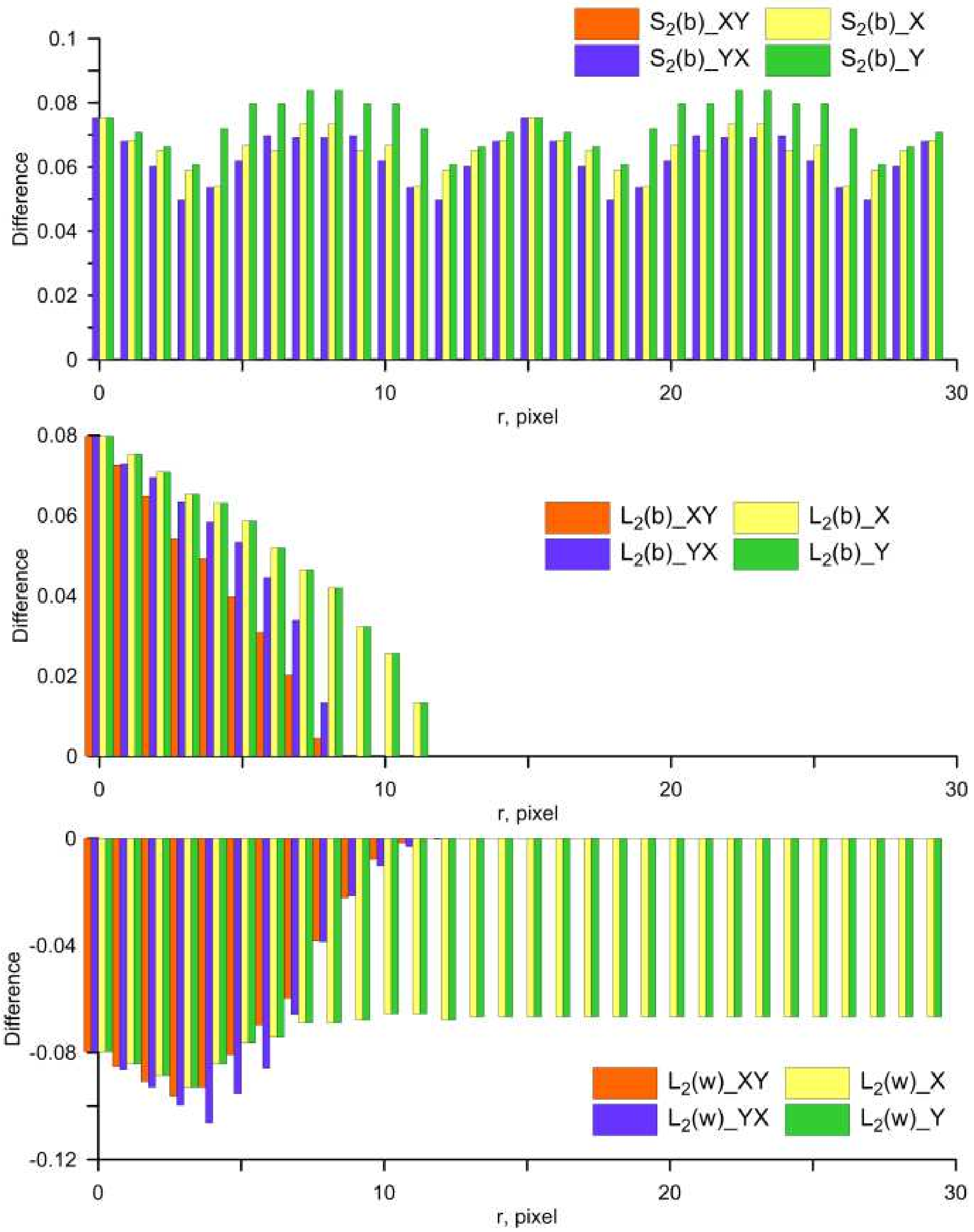

SI Figure 9 Difference between correlation functions computed for upscaled (coarsened resolution) image and upscaled correlation functions for original image. All notations are the same as for SIFig.8. Unlike previous case, direct image coarsening does not preserve the phase ratio any more. For images of porous media this would mean a porosity change which can significantly alter pore-scale modelling results.

SI5. Overall scheme of spatial data fusion for synthetic shale structure. All main steps performed to obtain a final fused image are shown in SIFig.10.

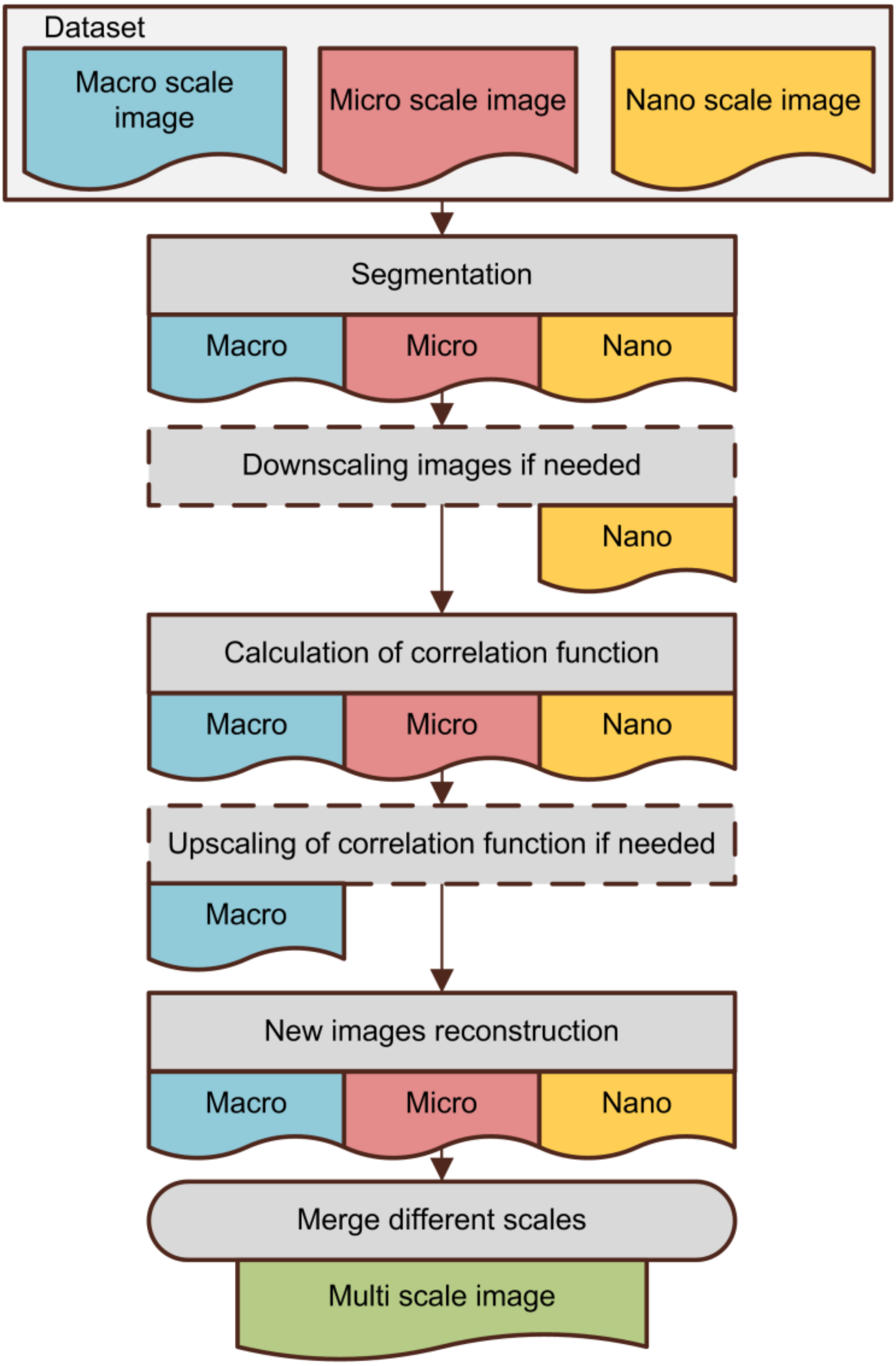

SI Figure 10 Workflow showing all major steps for merging macro, micro and nanoscale images.

SI.6 Computing resource specifications and computation times. All stochastic reconstructions were performed as described in Materials and Methods section of the paper. All simulations were single-threaded and performed on Intel Xeon X7560 2.26 GHz CPU. Our in-house code with efficient optimization of S_2 and L_2 functions recalculations at each annealing step is written in C++ and was executed under Windows environment. Compiling it under Linux could result in faster performance. The merging step was performed using the C# GUI program with the possibility to superimpose images in different order.

The loop of permutations and correlation function recalculations should be stopped at some point to finalize the reconstruction procedure. There are two popular ways to do that: 1) after some number of consecutive unaccepted permutations (10^6 in our case), or 2) by choosing some accuracy threshold value for the energy E. The latter method is preferable in routine reconstructions, as it will take much less time to reach an almost similar accuracy. However, the former explores the energy landscape more thoroughly and was utilized here, as the appropriate energy threshold is not known a priori. For this reason our reconstructions involving S_2-L_2 correlation functions were iterated for 3-5 days before such precise criterion was met. Reconstruction involving C_2 (nanoscale image) was iterated for two weeks. Resulting energies E were: 0.0000011570016282 for macro, 0.0000000059309015 for micro, and 0.0000002338290331 for nanoscale images.

Note that the efficiency of the computations depend on numerous parameters, among which the main are: 1) size of image being reconstructed (1024^2 for nanoscale and 4096^2 for two other images), 2) annealing cooling schedule parameters, for example, annealing schedule parameter λ (was 0.999999 in all our cases), 3) the spatial cut-off $|r|$ (values for all reconstructions performed here are available on SIFig.2-5) for the correlation lengths computed (requires to capture all correlation lengths on the original images, for that reason this value was very high in our reconstructions to reconstruct, for example, a very long horizontal inclusion on macroscale image). This all means that a direct measure of computational efficiency is not available. For the purpose of comparison we now report CPU time needed to perform 1000 permutations for macro, micro and nanoscale: 0.889, 0.499, and 1770.43 seconds, correspondingly (nanoscale image was reconstructed with the use of C_2 function which was not optimized and, thus, recalculated at each annealing step). Another measure of computational performance for images of around 1000^2 size can be found in our resent publication[6].

In total we made three reconstructions for a macroscale image, one reconstruction for microscale and two for nanoscale. The reconstruction quality for microscale was very good (visually and according to C_2). A first reconstruction of the nanoscale image involving only S_2-L_2 set resulted in too high connectivity for the pore phase and, thus, we reconstructed it involving the C_2 function as well (see details on SIFig.5). The microscale images' quality was good in all three cases, but due to periodic boundary conditions two of them were not appealing visually (the main horizontal white phase cluster appeared to be divided between upper and lower parts of the reconstructed image).

SI.7 Different phase embedding. To show the versatility of our method in dealing with numerous phases, we provide a simple manipulation with our reconstructions by variations in embedding strategies, as is evident from SIFig.11. Note that SIFig.11c represents the final fused multi-scale image obtained for our first synthetic shale rock example.

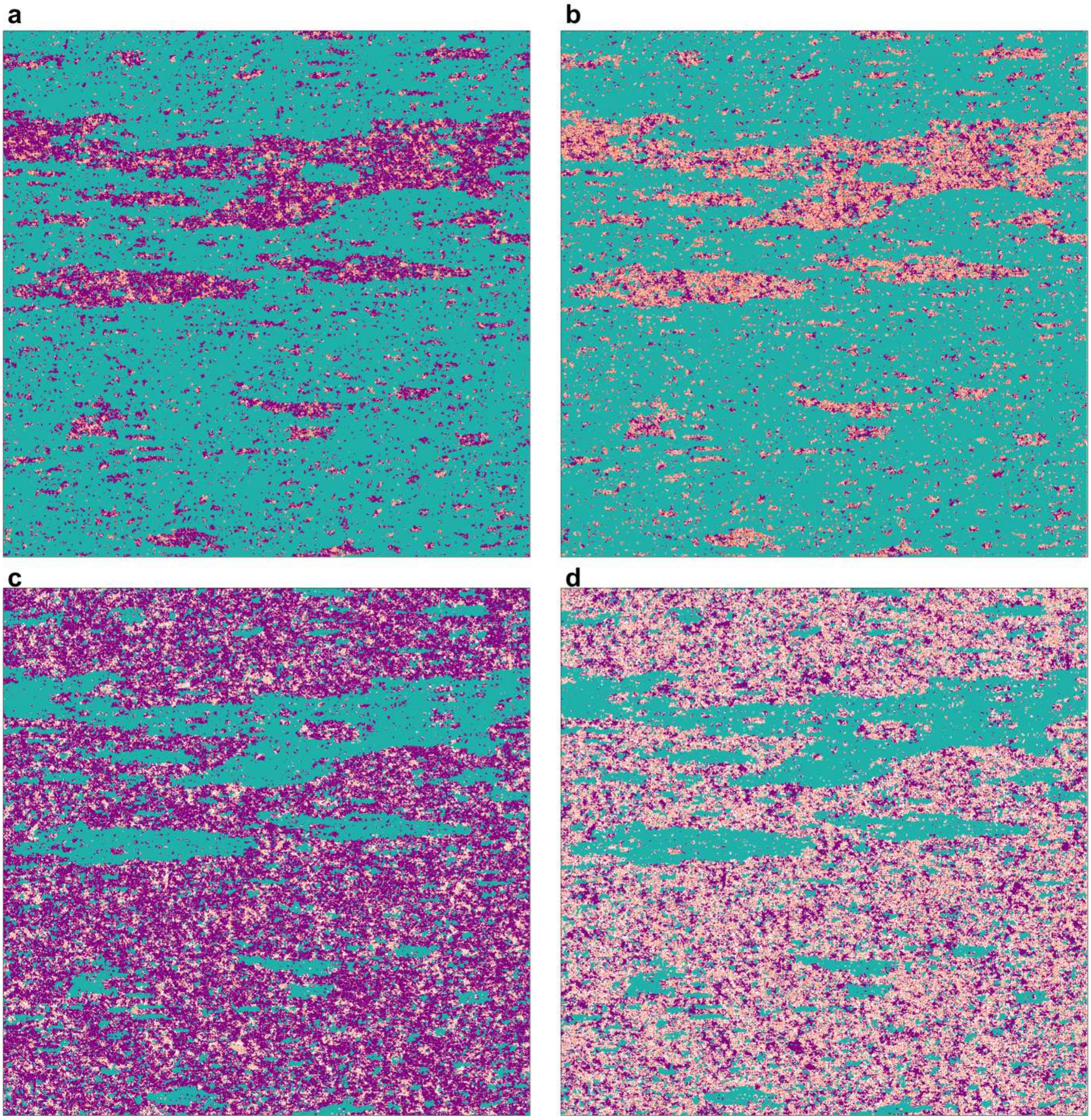

SI Figure 11 Different variations in embedding subsequent phases from three stochastic (macro, micro and nanoscale images) reconstructions.

SI8. Another realization of the fusing procedure where original macro-scale data is used. The results of merging the original magnified macroscale image and stochastic reconstructions for micro and nanoscale are show in SIFig.12.

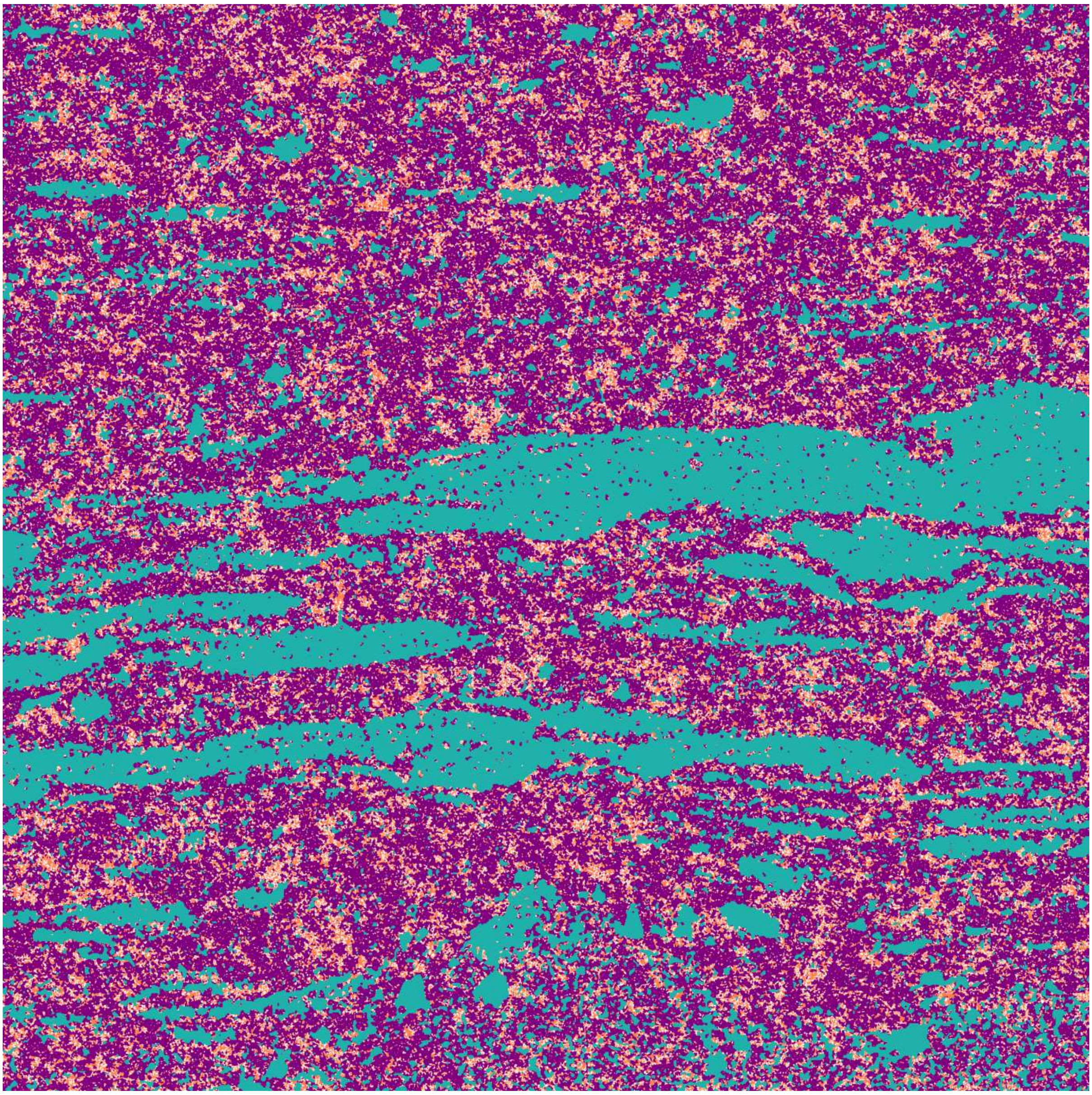

SI Figure 12 Fused image with original tomography image used to represent macroscale information during fusing. Phases are as follows: (i) resolved mineral (blue) and unresolved non-mineral phase (white) on macroscale image; (ii) mixture of resolved minerals and kerogen phases (purple) and unresolved kerogen with nano-pores (white) on microscale image; (iii) resolved kerogen (organic matter) phase (orange) and resolved kerogen nano-porosity (white) on nanoscale image.

SI9. Final multi-scale merged images in 4096^2 pixels size. Finally, we provide all full-size merged images as separate image files (note that for review process we provide Dropbox links, if this paper will be accepted we shall organize DOI link to CSIRO data storage):

1) https://dl.dropboxusercontent.com/u/101335355/-scale%20shale%20example.bmp – fused image for case one;
2) https://dl.dropboxusercontent.com/u/101335355/porosity.tif - fused multi-scale porosity image;
3) https://dl.dropboxusercontent.com/u/101335355/orig_macro_fused.bmp - fused image similar to case one, but using original macro-scale image during fusing step.

SI10. Supplementary References

1. Korost, D. V. , Nadezhkin, D. V., Akhmanov G. G. Pore space in source rock during the generation of hydrocarbons. *Moscow University Geology Bulletin* **67(4)**, 240-246 (2012).

2 Balushkina, N. S., Kalmykov, G. A., Belokhin, V. S. , Khamidullin, R. A. , Korost D. V. Siliceous reservoirs of the Bazhenov formation, the Sredny Nazym oil field, and the structure of their pore space. *Moscow University Geology Bulletin* **69(2)**, 91-100 (2014).

3. Gerke, K.M., Vasilyev, R.V., Korost, D.V., Karsanina, M.V., Balushkina, N., et al Determining Physical Properties of Unconventional Reservoir Rocks: from Laboratory Methods to Pore-Scale Modeling. *SPE 167058 Technical paper*, presented at SPE Unconventional Resources Conference and Exhibition, 11-13 November 2013, Brisbane, Australia. DOI: 10.2118/167058-MS (2013).

4. Gerke, K.M., Karsanina, M.V., Vasilyev, R.V., Mallants, D. Improving pattern reconstruction using directional correlation functions. *Europhys. Lett.* **106(6)**, 66002 (2014).

5. Rozman, M.G., Utz, M. Uniqueness of reconstruction of multiphase morphologies from two-point correlation functions. *Phys. Rev. Lett.* **89(13)**, 135501 (2002).

6. Karsanina M.V., Gerke K.M., Skvortsova E.B., Mallants D. Universal spatial correlation functions for describing and reconstructing soil microstructure. *PLoS ONE* **10(5)**, e0126515 (2015). DOI: 10.1371/journal.pone.0126515.
